# Surgical outcomes in Chiari malformation type I: A quality review from a Scandinavian medium-volume neurosurgical center

**DOI:** 10.1016/j.bas.2025.105919

**Published:** 2025-12-23

**Authors:** Einar Naveen Møen, Babisha Mathivannan, Rupavathana Mahesparan

**Affiliations:** a Department of Clinical Medicine, University of Bergen, Jonas Lies vei 91B, 5021, Bergen, Norway; b Department of Neurosurgery, Haukeland University Hospital, Haukelandsveien 22, 5021, Bergen, Norway

**Keywords:** Chiari malformation type 1, Posterior fossa decompression, 30-Day surgical quality indicators, Surgical outcomes, Complications, Hydrocephalus, Quality improvement

## Abstract

**Background:**

Chiari malformation type 1 (CM-I) is characterized by caudal herniation of the cerebellar tonsils through the foramen magnum. Surgical decompression is generally indicated in patients with significant or progressive symptoms, or in clinically relevant or progressing syringomyelia. As CM-I surgery is relatively infrequent in medium-volume neurosurgical centers, outcome data from such settings remain limited. This study evaluates surgical outcomes following CM-I decompression at a Scandinavian medium-sized neurosurgical center.

**Research question:**

What are the outcomes of CM-I surgery at the Department of Neurosurgery, Haukeland University Hospital?

**Material and methods:**

We performed a retrospective case series of patients with CM-I treated at our department between 2014 and 2023. The primary outcome was the Chicago Chiari Outcome Scale (CCOS) at the last follow-up. Secondary outcomes were 30-day surgical quality indicators.

**Results:**

Forty patients comprised of 29 (72.5 %) adults and 11 (27.5 %) pediatric patients. Clinical improvement (CCOS ≥13) was observed in 78 %. The observed 30-day quality indicators were reoperations in three patients (7.5 %), infections in two patients (5.0 %), and readmission in seven patients (18 %). Complications within 30 days occurred in six patients (15 %). The most frequent complication was new-onset hydrocephalus, which occurred in four patients (10 %). The median hospital length of stay was 5.5 days. There was no mortality.

**Discussion and conclusion:**

Most patients demonstrated clinical improvement, but complication rates exceeded benchmarks. Hydrocephalus was the most frequent issue. We discuss possible interventions to further strengthen CM-I care in our department, with an emphasis on hydrocephalus management.

## Highlights

• Clinical Improvement in Majority of Patients
• Radiological Success Despite Variable Clinical Response
• Hydrocephalus as Predominant Complication
• Proposal for Preoperative ICP Monitoring
• Quality Improvement Initiatives Introduced

## 1 Introduction

Chiari malformation is characterized by the displacement of the cerebellar tonsils through the foramen magnum. This may disrupt cerebrospinal fluid (CSF) flow at the foramen magnum and the fourth ventricle. Chiari malformations are classified into four types, differing fundamentally in morphology and severity. Chiari malformation type 1 (CM-I) is the most common (Friedlander, 2024). Radiological series report a prevalence of 0.5 %–3.6 %, though most cases are asymptomatic and discovered incidentally (Chumas et al., 2011; Birkmeyer et al., 2003; Halm et al., 2002). Typical symptoms include headaches exacerbated by activities increasing intracranial pressure, upper extremity paresthesia, and, less commonly, tinnitus, dysphagia and sleep apnea. Surgery is indicated when symptoms are significant or progressing, or if syringomyelia is clinically relevant or increasing. The goal of surgery is to restore CSF flow (Friedlander, 2024).

The Department of Neurosurgery at Haukeland University Hospital functions as the designated tertiary care referral center for Western Norway, providing 1.2 million with neurosurgical care. Surgical treatment of Chiari malformation represents a relatively rare intervention within the clinical portfolio of the department, with an average of 4–5 cases performed annually. These procedures are typically reserved for patients presenting with significant symptomatology such as characteristic headaches, radiological findings indicative of neural compression, syringomyelia, or cerebrospinal fluid flow obstruction.

The primary aim of this study was to evaluate surgical outcomes for Chiari malformation type 1 at our institution as part of an ongoing departmental quality assurance initiative. Expanding on this, we wanted to explore to what degree a medium-sized neurosurgical center can maintain high standards of care in CM-I surgery. This could contribute to the broader discussion on the volume-outcome relationship in rare neurosurgical conditions.

## 2 Methods and materials

### 2.1 Study design

This retrospective case series included patients who underwent surgical treatment for Chiari malformation type 1 at our department between 2014 and 2023. The primary outcome of our study was the Chicago Chiari Outcome Scale (CCOS) at the last follow-up. Secondary outcomes included 30-day quality indicators, including complication rates.

### 2.2 Data collection

Patients were identified from the hospital's operation registry, Lifecare Orbit Surgery Planning (Tietoevry, Finland). Data was collected on descriptive variables, comorbidities, symptoms at presentation, radiological examination, surgical technique, perioperative complications, postoperative complications, and 30-day quality indicators from electronic medical records and radiological reports. Comorbidities were assessed using the Charlson's Comorbidity Index. Mortality, readmissions, reoperations, infections, and complications occurring within 30 days postoperatively, and length of stay, were used as quality indicators. The CCOS was scored retrospectively by the authors through notes from follow-up consultations. Clinical improvement was defined as a ≥13-point score of CCOS. The first follow-up was typically conducted within 3–6 months. The end point was the last follow-up recorded in the patient's medical journal during the study period. No post-hoc patient interviews were conducted.

### 2.3 Surgical technique

The standard surgical procedure, posterior fossa decompression, includes suboccipital craniectomy laminectomy of the atlas (C1). The aim of surgery is to relieve pressure at the craniocervical junction and restore normal CSF flow. After completing the bony decompression, intraoperative ultrasound is used to assess the CSF space posterior to the cerebellar tonsils and visualize pulsations at the craniocervical junction. The decision to proceed with duraplasty depends on the intraoperative adequacy of CSF flow and tonsillar pulsation. This was performed in selected cases to enhance decompression and restore physiological flow of CSF. Alternatively, a slit durotomy, which avoids full duraplasty, was used to achieve the desired decompression. Duraplasty was routinely planned preoperatively in patients with syringomyelia, regardless of intraoperative ultrasound findings. For dural augmentation, a synthetic collagen implant (Lyoplant®, Aesculap AG, a B. Braun company) derived from lyophilized bovine pericardium was used.

### 2.4 Radiological evaluation

All patients selected for surgical treatment of Chiari malformation type I underwent standardized magnetic resonance imaging (MRI) preoperatively and postoperatively. Preoperative imaging included assessment of the degree of tonsillar herniation, craniocervical crowding, presence and extent of syringomyelia, and cerebrospinal fluid (CSF) flow dynamics using phase-contrast MRI. Postoperative imaging included an early MRI to evaluate adequacy of decompression and exclude complications, followed by repeat MRI prior to the first clinical follow-up, with reassessment of CSF flow dynamics and changes in syringomyelia.

### 2.5 Statistical analysis

Statistical analysis was performed using R version 4.3.3 in RStudio (RStudio, 2019; Team, 2025) with a significance level of α = 0.05. Descriptive analyses were performed using age group (pediatric versus adult with ≥18 years as a cut-off) as a stratification variable. The two groups were compared using Fisher's exact test for categorical variables with any expected cell counts <5, and Pearson's Chi-squared test for categorical variables with all expected cell counts ≥5, and Wilcoxon rank sum test for continuous variables.

### 2.6 Ethical considerations

The study protocol was exempt from review by the Regional Ethical Committee of Western Norway as it classified as a quality improvement study. Quality improvement studies only require permission from the local hospital. This arrangement is regulated by Norwegian law under the Personal Data Act (personopplysningsloven) article 6.1.e and 9.2.i, and the Specialist Healthcare Act (spesialisthelsetjenesteloven) §§ 3 – 4a.

## 3 Results

### 3.1 Patient population

Posterior fossa decompression was performed in 46 patients at our department over a period of 10 years (2014–2023). Three patients with Chiari malformation type 2, two patients with achondroplasia, and one patient where decompression was done as an emergency effort to relieve intracranial pressure, were excluded. This left us with 40 patients with CM-I comprised of 29 (72.5 %) adult patients with a median age of 36 years, and 11 (27.5 %) pediatric patients with a median age of 9 years. There was an overweight of women in the adult subgroup (24 [83 %]), and men in the pediatric subgroup (6 [55 %]) (p-value: 0.042), with 29 (72 %) women in total (Table 1). The median time to first follow-up was 106 days in total, 100 days for pediatric cases, and 107.5 days for adult cases. The median time to last follow-up was 408 days in total, 638 days for pediatric cases, and 229 days for adult cases.

**Table 1:** Baseline characteristics of 40 patients with Chiari malformation type 1 stratified by age group (pediatric <18 years vs. adult ≥18 years). Data presented as median (Q1, Q3) or n (%).

Characteristic	Total	Age Group		
	N = 40^a^	Pediatric N = 11^a^	Adult N = 29^a^	p-value^b^
Age at Surgery	28 (16, 38)	9 (6, 14)	36 (26, 42)	<0.001
Sex				0.042
Women	29 (73 %)	5 (45 %)	24 (83 %)	
Men	11 (28 %)	6 (55 %)	5 (17 %)	
Indication for Surgery				0.061
Chiari Headache	22 (55 %)	3 (27 %)	19 (66 %)	
Chiari Headache and Syringomyelia	1 (2.5 %)	0 (0 %)	1 (3.4 %)	
Head and Neck Spasms	1 (2.5 %)	0 (0 %)	1 (3.4 %)	
Syringomyelia	15 (38 %)	8 (73 %)	7 (24 %)	
Tinnitus	1 (2.5 %)	0 (0 %)	1 (3.4 %)	
Preoperative Imaging				0.2
Magnetic Resonance Imaging No Flow	30 (75 %)	10 (91 %)	20 (69 %)	
Magnetic Resonance Imaging Flow	10 (25 %)	1 (9.1 %)	9 (31 %)	

a Median (Q1, Q3); n (%).

b Wilcoxon rank sum test; Fisher's exact test.

During the same period, 120 patients with CM-I were evaluated for potential surgical treatment. Of these, only 30 % underwent decompression surgery. The most common surgical indication was functionally impairing headache attributed to CM-I. This was typically characterized by exacerbation during activities that increase intracranial pressure, such as coughing, sneezing, or straining, commonly referred to as Chiari headache. The second most common indication was syringomyelia (Table 1). A total of 27 patients underwent bony decompression with duraplasty, 10 underwent bony decompression only, and 3 underwent bony decompression with additional durotomy.

### 3.2 30-Day quality indicators and complications

The median length of hospital stay was 5.5 days. Three patients (7.5 %) required reoperation within 30 days because of new-onset hydrocephalus. A total of seven patients (18 %) were readmitted within 30 days. No 30-day mortality was observed (Table 2). Postoperative complications were recorded in six patients (15 %) (Table 3).

**Table 2:** Thirty-day quality indicators comparing pediatric and adult patients. Length of stay presented as median (Q1, Q3); other metrics as n (%).

Characteristic	Total	Age Group		
	N = 40^a^	Pediatric N = 11^a^	Adult N = 29^a^	p-value^b^
30-Day Length of Stay	5.50 (5.00, 7.00)	6.00 (5.00, 8.00)	5.00 (4.00, 6.00)	0.074
30-Day Mortality	0 (0 %)	0 (0 %)	0 (0 %)	
30-Day Readmission	7 (18 %)	4 (36 %)	3 (10 %)	0.075
30-Day Reoperation	3 (7.5 %)	2 (18 %)	1 (3.4 %)	0.2
30-Day Infections	2 (5.0 %)	0 (0 %)	2 (6.9 %)	>0.9
30-Day Complications	6 (15 %)	3 (27 %)	3 (10 %)	0.3

a Median (Q1, Q3); n (%).

b Wilcoxon rank sum test; Fisher's exact test.

**Table 3:** Detailed breakdown of 30-day complications by type and age group. Data presented as n (%).

Characteristic	Total	Age Group	
	N = 40^a^	Pediatric N = 11^a^	Adult N = 29^a^
30-Day New-Onset Hydrocephalus	4 (10 %)	3 (27 %)	1 (3.4 %)
30-Day CSF Leakage	3 (7.5 %)	1 (9.1 %)	2 (6.9 %)
30-Day Urinary Tract Infection	1 (2.5 %)	0 (0 %)	1 (3.4 %)
30-Day Surgical Site Infection	1 (2.5 %)	0 (0 %)	1 (3.4 %)

a n (%).

The most frequent complication was new-onset hydrocephalus, which occurred in four patients (10 %): three pediatric cases (7, 12, and 16 years) and one adult case (23 years). None of the patients exhibited clinical signs of hydrocephalus preoperatively, and ophthalmological examinations were normal. Retrospective review of MRIs revealed slight ventriculomegaly in patients C and D, and a colloid cyst without obstruction in patient B. All patients developed communicating hydrocephalus postoperatively. Patients B, C, and D required ventriculoperitoneal (VP) shunt placement, while hydrocephalus in patient A resolved after transient lumbar drainage (Fig. 2).

CSF leakage occurred in three patients. Two of these also developed hydrocephalus (patient B and D) and required shunt placement, while the third patient was managed with temporary lumbar drainage. Notably, all cases of hydrocephalus and CSF leakage occurred in patients who had undergone duraplasty. Two patients were readmitted due to headache, which resolved with conservative pain management. Two postoperative infections were documented: one urinary tract infection in a patient with new-onset hydrocephalus, and one surgical site infection in a patient with concurrent CSF leakage (Table 3).

### 3.3 Treatment outcomes

**Fig. 1:**
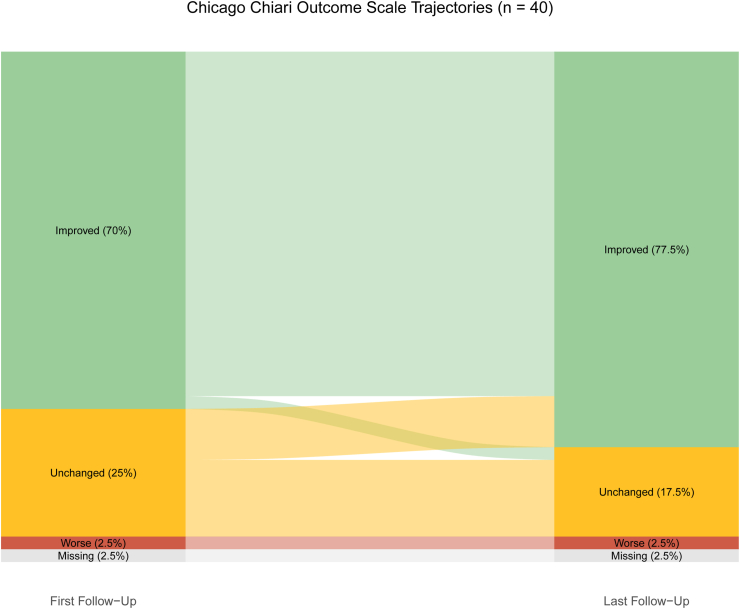
Alluvial diagram showing CCOS outcome trajectories from first to last follow-up. Improved defined as CCOS ≥13 points.

Clinical improvement (CCOS ≥13) was observed in 28 patients (70 %) at the initial follow-up. This number increased to 31 patients (78 %) at the final follow-up. Pediatric cases demonstrated higher improvement rates (82 %) compared with adults (76 %) at the final follow-up, but this difference was not statistically significant. The mean CCOS score at the last follow-up was 13.8, with subgroup means of 14.0 in pediatric patients and 13.7 in adults (Fig. 1, Table 4). Improved CSF flow or space around the cerebellar tonsils was observed in 38 patients (95 %). In the 22 patients with syringomyelia, a reduction in the syrinx diameter was seen in 14 patients (64 %) at the first follow-up, which increased to 21 patients (95 %) at the last follow-up. Data on follow-ups was not available in one patient (Table 4).

**Fig. 2:**
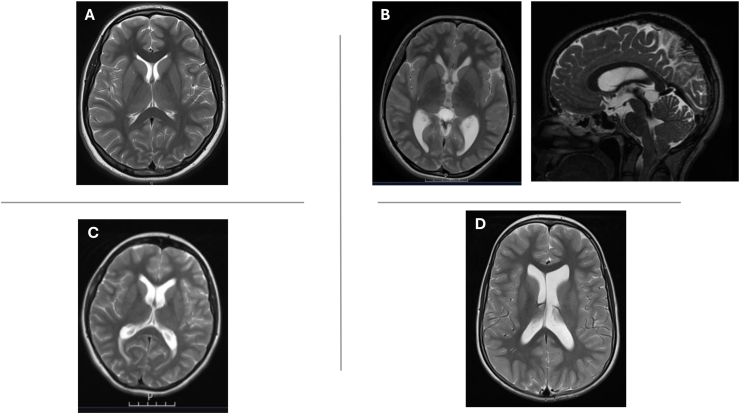
Preoperative MRI scans of four patients who later developed postoperative communicating hydrocephalus following posterior fossa decompression surgery. None of the patients exhibited clinical signs of hydrocephalus preoperatively. Retrospective imaging review revealed subtle findings such as borderline ventriculomegaly (B, C and D) suggesting altered CSF dynamics.

**Table 4:** Chicago Chiari Outcome Scale (CCOS) scores and patient-reported outcomes at first and last follow-up. CCOS scores presented as mean (SD); categorical outcomes as n (%).

Characteristic	Total	Age Group		
	N = 40^a^	Pediatric N = 11^a^	Adult N = 29^a^	p-value^b^
Chicago Chiari Outcome Scale Average at First Follow-Up	13.44 (2.47)	13.09 (2.63)	13.57 (2.44)	0.6
Missing	1	0	1	
Chicago Chiari Outcome Scale Distribution at First Follow-Up				0.7
Improved	28 (70 %)	7 (64 %)	21 (72 %)	
Unchanged	10 (25 %)	4 (36 %)	6 (21 %)	
Worse	1 (2.5 %)	0 (0 %)	1 (3.4 %)	
Missing	1 (2.5 %)	0 (0 %)	1 (3.4 %)	
Chicago Chiari Outcome Scale Average at Last Follow-Up	13.77 (2.10)	14.00 (1.55)	13.68 (2.29)	>0.9
Missing	1	0	1	
Chicago Chiari Outcome Scale Distribution at Last Follow-Up				>0.9
Improved	31 (78 %)	9 (82 %)	22 (76 %)	
Unchanged	7 (18 %)	2 (18 %)	5 (17 %)	
Worse	1 (2.5 %)	0 (0 %)	1 (3.4 %)	
Missing	1 (2.5 %)	0 (0 %)	1 (3.4 %)	

a Mean (SD); n (%).

b Wilcoxon rank sum test; Fisher's exact test.

## 4 Discussion

Only a small proportion of those with radiological CM-I require surgery (Leon et al., 2019; Davidson et al., 2021). Symptoms are often nonspecific, and imaging alone does not reliably predict surgical need. This makes patient selection challenging – particularly in medium-volume centers. At our center, we reserve intervention for patients presenting with significant symptomatology with radiological findings indicative of cerebrospinal fluid flow obstruction or syringomyelia that is symptomatic or progressive. During the study period, 40 of 120 referred patients were deemed surgical candidates. This suggests an intervention threshold consistent with international practice. For rare conditions like CM-I where indications are nuanced, clinical judgment and careful selection may be as important as procedural volume.

Our clinical improvement rate of 78 %, comprised of 76 % in adult cases and 82 % in pediatric cases, appears to fall within the range reported by other centers using the same outcome scale. Adult series with 107–318 patients report improvement rates from 62.5 % to 90 % (Kristiansson et al., 2022; Hernández-Hernández et al., 2024; Wang et al., 2023). One pediatric series with 71 cases found an improvement rate of 96 % (El-Hajj et al., 2024). Another pediatric series of 255 cases found a mean CCOS score of 14.6 (Mazerand et al., 2022).

Although not all patients in our cohort experienced clinical improvement as measured by the Chicago Chiari Outcome Scale (CCOS), radiological findings were notably positive in the vast majority of cases (95 %). Specifically, restored cerebrospinal fluid (CSF) flow at the craniocervical junction and resolution of syringomyelia were consistently observed postoperatively. This discrepancy between radiological success and clinical outcome suggests that factors beyond anatomical correction may influence patient recovery. Significant life events, psychological stressors, or comorbid conditions could potentially contribute to persistent symptoms among non-responders. These findings underscore the importance of a holistic approach to postoperative evaluation, integrating both imaging and patient-reported outcomes to better understand and address the multifactorial nature of recovery.

Complications arose in 6 (15 %) patients in our cohort. This is higher than the rate reported in the literature, including those reported in a registry-based study with 1947 operations (14.3 %). Postoperative new-onset hydrocephalus occurred in four patients (10 %), making it the most frequent complication in our series. Reported rates of postoperative hydrocephalus in CM-I range from 5 % to 7 % (Bartoli et al., 2020). Postoperative hydrocephalus is a recognized complication after posterior fossa decompression, even in patients without preoperative signs of raised intracranial pressure (ICP) (Zakaria et al., 2012). In our series, all four patients were asymptomatic, yet retrospective MRI revealed subtle findings such as ventriculomegaly and a colloid cyst, suggesting altered CSF dynamics. This raises the question of whether intracranial pressure monitoring could improve risk stratification and perioperative management.

Chiari I malformation (CM-I) and hydrocephalus share a complex, bidirectional relationship. Hydrocephalus may cause tonsillar descent, while tonsillar herniation can obstruct CSF flow (Piper et al., 2015). ICP monitoring can help distinguish these mechanisms and guide treatment – avoiding unnecessary decompression when CSF diversion might suffice. Frič et al. reported that abnormal intracranial compliance, reflected by elevated pulse pressure amplitude, was more common than raised mean ICP in CM-I patients. This indicates that standard ICP thresholds may miss clinically relevant changes (Frič et al., 2023, 2024). Preoperative overnight ICP monitoring could help identify patients with altered intracranial compliance, who may be at a higher risk of postoperative hydrocephalus (Duy et al., 2024). This may inform perioperative management by prompting earlier postoperative imaging and a lower threshold for intervention, should symptoms of hydrocephalus emerge. However, who should undergo such assessment, and when, remains undefined. Prospective studies are needed to determine whether preoperative ICP monitoring can identify patients at risk for postoperative hydrocephalus in CM-I surgery.

Physiologically, increased CSF volume or venous congestion elevates ICP, potentially worsening tonsillar descent (Monro-Kellie principle) (Mokri, 2001). Recognizing this interplay underscores the role of ICP monitoring not only postoperatively but also preoperatively in selected CM-I cases. We hypothesize that patients with borderline ventricular enlargement or ambiguous imaging may benefit from ICP monitoring before decompression. This approach could reduce postoperative hydrocephalus and shunt dependency. Future studies should define criteria for ICP assessment and evaluate its impact on surgical outcomes.

The underlying pathophysiology for postoperative hydrocephalus is likely multifactorial, with disrupted cerebrospinal fluid (CSF) dynamics at the craniocervical junction as a common contributing factor. A previous retrospective case series identified younger age (<6 years), increased intraoperative blood loss, and the presence of a fourth ventricular web as potential risk factors for postoperative hydrocephalus (Guan et al., 2016). In our cohort, no specific risk factors for the development of hydrocephalus were identified, apart from the fact that all affected patients had undergone duraplasty and three were pediatric cases. These variables will be considered closely in future preoperative planning at our department.

The second most common complication in our series was CSF leakage, with a prevalence of 7.5 %. Two cases were associated with hydrocephalus and treated with VP shunts, and one required reoperation with dural repair. The prevalence of CSF leakage after CM-I surgery is uncertain and varies widely in the literature. One study found a prevalence of patients requiring reoperation for CSF leakage of 4 % (Bhimani et al., 2018). The prevalence likely depends on surgical technique, patient-specific factors, and dural graft material (Bhimani et al., 2018). In our cohort, postoperative development of hydrocephalus appeared to contribute to CSF leakage, as this occurred in two of the three affected cases. Increased intracranial pressure, impaired wound healing, and a longer operating corridor have been proposed as explanations for this phenomenon (Bhimani et al., 2018). These mechanisms are associated with an increased body-mass index, and could explain why obese patients are at a greater risk of complications (Bhimani et al., 2018). This is compounded by obesity increasing the risk of developing CM-I and syringomyelia (Arnautovic et al., 2013, 2015). Intraoperatively, reinforced dural closure using grafts, and dural sealants may help reduce the risk of leaks.

In a generalized effort to further improve our results, we will establish a local prospective quality registry for rarely encountered disorders (<10 cases annually) at our department. This registry will include Chiari malformations, utilizing CCOS as a validated assessment tool alongside traditional 30-day quality indicators (Aliaga et al., 2012). Systematic performance evaluation through this registry will enable identification of adverse event patterns and targeted quality improvement interventions. Additionally, we will create a multidisciplinary team for rare procedures, meeting monthly to facilitate systematic planning and discussion of individual cases.

## 5 Limitations

Measurement bias may have occurred given our reliance on retrospectively reviewing electronic patient records, despite efforts to minimize this through standardized variable definitions. Surgical indications and treatment algorithms likely vary between different neurosurgical centers, creating selection bias and limiting generalizability. The limited number of cases results in imprecise estimates, limiting generalizability and precluding reliable subgroup comparisons. This is compounded by using different techniques, as outcomes of posterior fossa decompression with and without duraplasty differs (Kristiansson et al., 2022; Lin et al., 2018).

## 6 Conclusion

Most patients in our cohort experienced lasting improvement and effective radiological decompression. This demonstrates that Chiari malformation type 1 surgery at a medium-volume Scandinavian neurosurgical center can yield clinical outcomes comparable to those reported by higher-volume institutions. However, the higher than expected rate of postoperative hydrocephalus shows a need for targeted quality improvement.

To improve our care, our department will implement several quality assurance initiatives. Preoperative monitoring of ICP may help identify patients at risk for postoperative hydrocephalus, particularly those with borderline ventricular enlargement or ambiguous imaging. We also plan to establish a structured clinical registry for rare neurosurgical conditions, integrating surgical outcomes and patient-reported measures. Together with a dedicated multidisciplinary team for complex, low-volume procedures, these efforts aim to support individualized care, improve outcome tracking, and foster continuous learning and improvement.

## Author contributions

**Conceptualization**: RM, BM, and ENM. **Formal analysis**: ENM. **Investigation**: RM, BM, and ENM. **Methodology**: ENM and RM. **Project administration**: RM. **Supervision**: RM. **Validation**: RM. **Visualization**: ENM. **Writing – original draft**: ENM and BM. **Writing – review and editing**: ENM, RM, and BM.

## Funding sources

None.

## Declaration of competing interest

The authors whose names are listed below certify that they have no affiliations with or involvement in any organization or entity with any financial interest (such as honoraria; educational grants; participation in speakers’ bureaus; membership, employment, consultancies, stock ownership, or other equity interest; and expert testimony or patent-licensing arrangements), or non-financial interest (such as personal or professional relationships, affiliations, knowledge or beliefs) in the subject matter or materials discussed in this manuscript.

## Data Availability

The data collected and analyzed in this study contains patient information and will therefore not be made publicly available.
